# 'At least there is something in my bra': A qualitative study of women's experiences with oncoplastic breast surgery

**DOI:** 10.1111/jan.15309

**Published:** 2022-07-07

**Authors:** Stine Thestrup Hansen, Lene Anette Willemoes Rasmussen

**Affiliations:** ^1^ Department of Plastic and Breast Surgery Zealand University Hospital Roskilde Denmark; ^2^ Department of Regional Health Research University of Southern Denmark Odense Denmark

**Keywords:** advanced nursing, breast cancer, longitudinal research, oncoplastic breast surgery, recovery, supportive care needs

## Abstract

**Aims:**

This study explores how women diagnosed with breast cancer may be supported by physicians and nurses during physical and existential changes related to oncoplastic breast surgery in Denmark. The following research questions were addressed: (a) how do women experience oncoplastic breast surgery, and (b) how does cancer treatment affect their body image?

**Design:**

A descriptive qualitative study design with a six‐step thematic analysis influenced by Braun and Clarke was applied in this study. This paper has been prepared in accordance with the consolidated criteria for reporting qualitative research.

**Methods:**

Fourteen in‐depth interviews with seven women diagnosed with breast cancer were conducted from August 2018 to March 2019. In this qualitative study, data analysis was performed concurrent with data construction, recognizing that the process of analysis and making sense of data should start during the interviews. We explicitly frame the discussion of the findings in a theory of embodiment influenced by Merleau‐Ponty, consistent with the construct of exploring human experiences to generate meaningful knowledge for applied practice.

**Results:**

Two overall themes with related subthemes were identified: (1) 'Treatment is required for life‐threatening cancer', and (2) 'Striving for a new normal body'. Across both themes, women's experiences reflected a 'time pendulum' as they contemplated their past identity, their current rationale and their transition to a future beyond breast cancer with a changed body.

**Conclusion:**

Participants reflected on their past, present and future when facing an altered body image caused by their breast cancer diagnosis and oncoplastic breast surgery. The participants in the study expressed broad levels of satisfaction with the results of the oncoplastic breast surgery. The reconstructed breast helped them to live normally again, in particular maintaining interpersonal relationships. Breast reconstruction supported participants' embodiment experiences and redefinition of their 'new normal'.

**Impact:**

This study showed the dynamic changes in self‐definition from receiving a breast cancer diagnosis and cancer treatment to oncoplastic breast surgery. The main finding of self‐redefinition was from the perspective of breast cancer women who were in a period of transition between post‐diagnosis and consultation for oncoplastic breast surgery. The findings indicate that advanced nurse specialists in the field of oncoplastic breast surgery can enhance psychosocial wellbeing and support women pre‐ and post‐operatively by focusing on patient experiences of self‐image and embodiment.

## 1 INTRODUCTION

Women diagnosed with breast cancer in Western countries are increasingly offered oncoplastic breast surgery as part of breast cancer treatments. Formerly, the primary focus of breast cancer treatment was lumpectomy or mastectomy, with little to no focus on postsurgical aesthetics or psychosocial aspects related to the process and its result. However, the number of breast cancer survivors continues to grow due to advancements in surgical and medical treatment. Therefore, long‐term outcomes and the experiences of individuals with breast cancer, such as quality of life related to body image and satisfaction, have become increasingly important components of breast cancer treatment and rehabilitation. Moreover, the development of surgical techniques such as microsurgery offers more opportunities and solutions in oncoplastic breast surgery (Macmillan & McCulley, 2016). Contemporary, state‐of‐the‐art aesthetic goals for oncoplastic breast surgery have therefore evolved to include the retention or restoration of one or both breasts to near normal shape, appearance, symmetry and size following breast cancer surgery. Standards for oncoplastic breast surgery are now described in a European guide on best practices (Gilmour et al., 2021). In Denmark, the Danish Breast Cancer Cooperative Group and the Danish Health Authority provide guidelines on best practices for breast cancer for accelerated inquiry, diagnostics and treatment, including oncoplastic breast surgery, if relevant (The Ministry of Health, 2018).

Previous research indicates that women who undergo breast reconstruction after breast cancer treatment report the highest long‐term satisfaction with their breasts, with a lower incidence of depression, while women undergoing mastectomy without reconstruction report the lowest satisfaction (Atisha et al., 2015). This could indicate that reconstruction should be recommended for all women diagnosed with breast cancer. However, although nurses advocate for scientific evidence about care, health and illness, they also know that the standardizing tendencies of evidence‐based practice can overrule individual variation, cultural needs, preferences and rights. In nursing theory, bodily changes are not simply objective goals, they are also subjective experiences that influence the individual's experience of the body in the world and the lived life. Furthermore, individuals are challenged and confronted with the fact that bodies are individually experienced but culturally and socially produced (Hopwood & Hopwood, 2019a), meaning that women's bodily experiences might be too complex and individual a phenomenon to capture through simple satisfaction outcomes.

During clinical practice, nurses meet women recently diagnosed with breast cancer who struggle with complex decisions about whether to choose oncoplastic breast surgery or conventional breast cancer surgery. Women are usually offered one of three equally effective oncologic surgical strategies: breast‐conservation surgery, mastectomy or mastectomy with breast reconstruction (and contralateral symmetry surgery, when relevant). The care and treatment of women undergoing these surgeries require nurses to consider issues related to body image and other psychosocial aspects. This qualitative study investigated experiences of Danish women diagnosed with breast cancer who received oncoplastic breast surgery to learn how nurses can support women during such bodily and existential changes.

### 1.1 Background

The prognosis for breast cancer has significantly improved in recent decades: currently, over 1 million women worldwide are diagnosed with breast cancer annually, and there is a 75% survival rate in most developed countries (World Health Organization, 2021). As a result, cosmetic concerns after breast cancer treatment affect an increasing number of women, including scars, large excision volumes, breast asymmetry, breast shape change, nipple displacement, scar retraction, skin alterations and radiation boost that can negatively impact outcomes in addition to the issues previously listed (Everaars et al., 2021; Ozmen et al., 2015). Further, poor cosmetic results may negatively impact body image and self‐esteem, or result in impaired sexuality or depression (Negenborn et al., 2017). Emphasizing cosmetic results may decrease psychological distress and improve the quality of life in breast cancer survivors (Hau et al., 2013; Kim et al., 2015).

Women diagnosed with breast cancer face different options for treatment, including surgery, chemotherapy, irradiation and hormonal therapy. In Denmark, multidisciplinary tumour management conferences provide recommendations for treatment at regular meetings comprising a team of healthcare specialists, including pathologists, oncologists, breast surgeons, plastic surgeons and registered breast cancer care nurse coordinators, all of whom are involved at different stages of a patient's cancer management plan. Based on a review of individual patients, this team reaches a consensus on suggested treatment. Treatment recommendations are based on tumour pathology, cancer stage, indications of potential metastasis and patient status, to achieve positive oncological outcomes and acceptable aesthetic results (Gilmour et al., 2021; Prades et al., 2015), and may include oncoplastic breast surgery and contralateral symmetry surgery. Oncoplastic breast surgery is a fairly new technique that combines plastic surgery with surgical oncology. In a typical procedure, the tumour is removed and the remaining breast tissue is reshaped or expanders are implanted to achieve symmetry and a more natural breast appearance (Berry et al., 2010). Oncoplastic breast surgery reduces (a) the time required to adjust to a reconstructed breast and altered body image (typically, this takes 1 year or more), including potential impacts on quality of life, emotional well‐being and intimacy; and (b) the range of physical and psychological impacts of surgery (e.g., discomfort, lack of sensation, self‐consciousness, body image issues) that may contribute to dissatisfaction with outcome (Gilmour et al., 2021). Body image is a key motivation for oncoplastic breast surgery. However, the procedure is more complex than standard breast cancer surgery and requires either both a breast surgeon and a plastic surgeon to carry out the operation, or one surgeon with oncoplastic certification, which may delay the patient's cancer management plan. Significant delays to breast cancer surgery may be associated with an increased risk of mortality (Hanna et al., 2020). Furthermore, oncoplastic breast surgery often requires volume displacement or volume replacement techniques, with contralateral symmetry surgery as required (Rizki et al., 2013).

The term body image relates to the concept of body and mind and has been developed and characterized by different historical and epistemological approaches. Modern dualistic approaches can be traced back to René Descartes (1596–1650), who saw human beings as consisting of two parts, body and mind, implying a mechanical perception with no causal connection between the two parts (Cash & Pruzinsky, 2002). In contrast, in 1944, the phenomenologist Maurice Merleau‐Ponty described the concept of the mind and body as a whole (Merleau‐Ponty, 2011). In 1990, Bob Price, a nurse, developed the body image care model (Price, 1990), which focuses on how humans experience bodily changes as a result of illness, injury or disability. The model describes body image as influenced by three dimensions: body ideal, body reality and body appearance.

From an anthropological perspective, the body is viewed as a product of a specific historical, social and cultural context that influences how a person interacts with the surrounding world. The literature increasingly recognizes that the interface between the physical, psychological and social realms matters for body image. Patients may be affected by medical illness and treatment, leading to an altered body, changes in bodily functions and the personal and social consequences of these changes (Hopwood & Hopwood, 2019b; Rezaei et al., 2016).

The results from previous research on body image following treatment for breast cancer are diverse. Most studies concentrate on women's experiences related to body image after either mastectomy or lumpectomy (Brunet et al., 2013; Collins et al., 2011). These studies find that overall, women experience physical changes in a mostly negative way, which shapes their perceptions, thoughts, attitudes, feelings and beliefs about their bodies. Other research finds that discomfort related to body image decreases over time regardless of the type of breast surgery, if comparing breast‐conserving surgery, mastectomy alone and mastectomy with reconstruction (Collins et al., 2011). Furthermore, research also shows that mastectomy is associated with more body image issues compared with lumpectomy and mastectomy with reconstruction, and that women who have had reconstruction experience higher satisfaction with their sexual life and with the aesthetic and cosmetic result (Marinkovic et al., 2021). In addition, these women tend to experience less embarrassment related to their body and less negative attention related to the disease (Fernández‐Delgado et al., 2008). Women who choose reconstruction also tend to experience a balanced body image and expect to retain a sense of femininity, both physically and mentally (Collins et al., 2011; Crompvoets, 2006; Snöbohm et al., 2010). Other research indicates that women's experiences of body image can be subjective and dependent on multiple individual factors, such as context, culture, social relations, cancer severity, tumour size, stage and volume of excision, among others (Gilmour et al., 2021; Negenborn et al., 2017). In summary, a growing body of literature evidences that the experience of breast cancer seriously affects women and their overall sense of body image (Boquiren et al., 2013; Jabłoński et al., 2019) for years after diagnosis and treatments (Dahl et al., 2010).

In the care and treatment of women diagnosed with breast cancer, hospital nurses and physicians still tend to evidence a dualistic understanding of the body, focusing more on treatment regimens and less on the whole person (Paraskeva et al., 2019; Schmid‐Büchi et al., 2008). The complexity of body image presents an ongoing challenge to better recognize and understand the diverse issues underpinning it, including the extent and causes of, and recovery from, body image disruption (Hopwood & Hopwood, 2019b). The available research and knowledge on body image after oncoplastic breast surgery is sparse, as most research has investigated body image using scale‐based questionnaires rather than individual self‐expression. Furthermore, little is known about the information needed by women receiving oncoplastic breast surgery, or their expectations.

## 2 THE STUDY

### 2.1 Research questions and aims

This study aimed to investigate how women diagnosed with breast cancer may be supported by nurses during bodily and existential changes related to oncoplastic breast surgery. The following research questions were addressed: (a) how do women experience oncoplastic breast surgery over time, and (b) how does cancer treatment affect their body image over time?

### 2.2 Design

The design of this study was influenced by the authors' and their nursing colleagues' experiences in a breast surgery outpatient clinic. Nurses reported that physicians advocated for oncoplastic surgery to provide improved aesthetic results after breast cancer and thereby achieve an improved quality of life in the long term, as showed in research (Atisha et al., 2015). However, nurses noted that patients' levels of satisfaction related to their breast(s) were more associated with body image, acceptance of the oncoplastic surgery, and postoperative pathways. Therefore, the authors assumed that women's perspectives on experiences of body image over time, both during and after oncoplastic cancer treatment, could provide insights to inform future practice. To explore this, a descriptive qualitative study design with a thematic analysis influenced by Braun and Clarke was chosen for its capacity to explore women's experiences (Braun et al., 2013), and individual in‐depth interviews with key informants were collected. Individual interviews are ideally suited to explore experiential research questions and can also be useful for exploring understandings and perceptions (Braun & Clarke , 2013). The study was conducted by two experienced female nurse researchers employed at the same department. The first author is a registered nurse, Master of Science in Nursing and PhD, while the second author is a nurse specializing in clinical practice with more than 10 years of experience in applied research. An EQUATOR checklist for reports of interviews and a COREQ focus group studies checklist were applied (Tong et al., 2007); see further details in Appendix B.

### 2.3 Participants and settings

Participants were recruited face to face by the second author in August and September 2018 at a large breast surgery outpatient clinic at a Danish university hospital, during patient consultation appointments. At the time of the interviews, the department of plastic surgery and the department of breast surgery were separate departments located at different hospitals in the region. This was a limitation for the speciality, complicating interprofessional collaboration between the departments involved in the treatment and care of women diagnosed with breast cancer. In 2015, the departments were merged into the Department of Plastic and Breast Surgery but were not physically consolidated at the same hospital location until 2020.

In total, nine candidates for oncoplastic breast surgery were invited to participate in the interviews. Two women declined to participate. The participants were therefore seven female Danish‐speaking patients diagnosed with breast cancer or ductal carcinoma in situ who had chosen oncoplastic breast surgery. All participants had proactively requested oncoplastic breast surgery, which was not a standardized treatment at the time of the study due to limited collaboration between the specialities. Participants were purposefully sampled for maximum variation of demographic characteristics, providing variability of participant experiences with respect to the phenomenon explored. Participant demographics and other variables are presented in Table 1.

**TABLE 1:** Participant variables and categories

Variable	Categories	Frequency (*f*)
Age	30–39 years	1
	40–49 years	2
	50–59 years	4
Number of children	0	1
	2	3
	3	3
Education	Diploma	2
	Bachelors degree	4
Relationship status	Married	6
	Co‐habiting	1
Diagnosis	Breast cancer	6
	Ductal carcinoma in situ	1
Treatment modality (>1 method is possible)	Breast conservation surgery	1
	Mastectomy with breast reconstruction	6
	Contralateral balancing	1
	Bilateral breast reduction	1
	Chemotherapy	6
	Irradiation	2
	Hormonal therapy	4
(*N* = 7)		

In Denmark, all facilities treating breast cancer are part of the multidisciplinary Danish Breast Cancer Cooperative Group, which prepares evidence‐based guidelines about diagnostic procedures, treatment and follow‐up to improve prognosis (The Danish Breast Cancer Cooperative Group [DBCG], n.d.). The healthcare system is government‐funded for all citizens, indicating that the population served by any given treatment centre represents all patients regardless of socioeconomic background.

### 2.4 Data collection

The interviews were conducted by the second author from August 2018 to March 2019 as in‐depth interviews following a semi‐structured interview guide (Braun & Clarke, 2013) influenced by Price's theoretical constructs of body ideal, body reality and body presentation, on the assumption that an altered body image affects women's experiences (Price, 1990). The interview guide is presented in Appendix A. Participants met individually with the second author in a conference room in the hospital and were explained the research purpose and participants' rights. Each participant was interviewed twice face‐to‐face to investigate personal development and experiences over 6 months, resulting in a total of 14 interviews. The initial interview was conducted after each participant's oncoplastic breast surgery, in 30 days of diagnosis. The second interview was conducted approximately 6 months after the first interview. Each interview lasted 30–45 min. The interviews were audio‐recorded, transcribed verbatim and uploaded to the qualitative software programme NVivo for analysis.

### 2.5 Data analysis and rigour

For rigorous qualitative sampling and saturation of data, Braun et al. (2013) propose that qualitative researchers require a sample that is appropriate to the research question and the theoretical aims of the study, and which can provide an adequate amount of data to fully answer the question and analyse the issue. After the 14 interviews, the authors concluded that the interview guide questions were rigorous enough for a substantial thematic analysis and sufficient to lead to relevant findings.

Interviews and analysis were initially guided by the research questions. However, as the process of data collection and interpretation evolved, the theoretical framework was adapted. The theoretical framework for the present study was influenced by Merleau‐Ponty's phenomenology of perception and embodiment, consistent with the construct of exploring human experiences and producing meaningful knowledge for applied practice. Merleau‐Ponty argues that human understanding comes from our bodily experience of the world that we perceive (Merleau‐Ponty, 2011). He proceeds from the assumption that human existence is bodily and that we as subjects are embodied, meaning inseparable from our bodies and our world. He defines body image as 'the ways meanings, expectations, and habits are experienced and expressed in the body' (Merleau‐Ponty, 2011). Thus, illness affects the whole person and its result is not simply objective, e.g., a reconstructed breast. Rather, there is a complex relationship between meaning, body, and diagnosis and women diagnosed with breast cancer have their own perceptions of the illness. This approach enabled us to understand illness experiences from a first‐person perspective.

**FIGURE 1:**
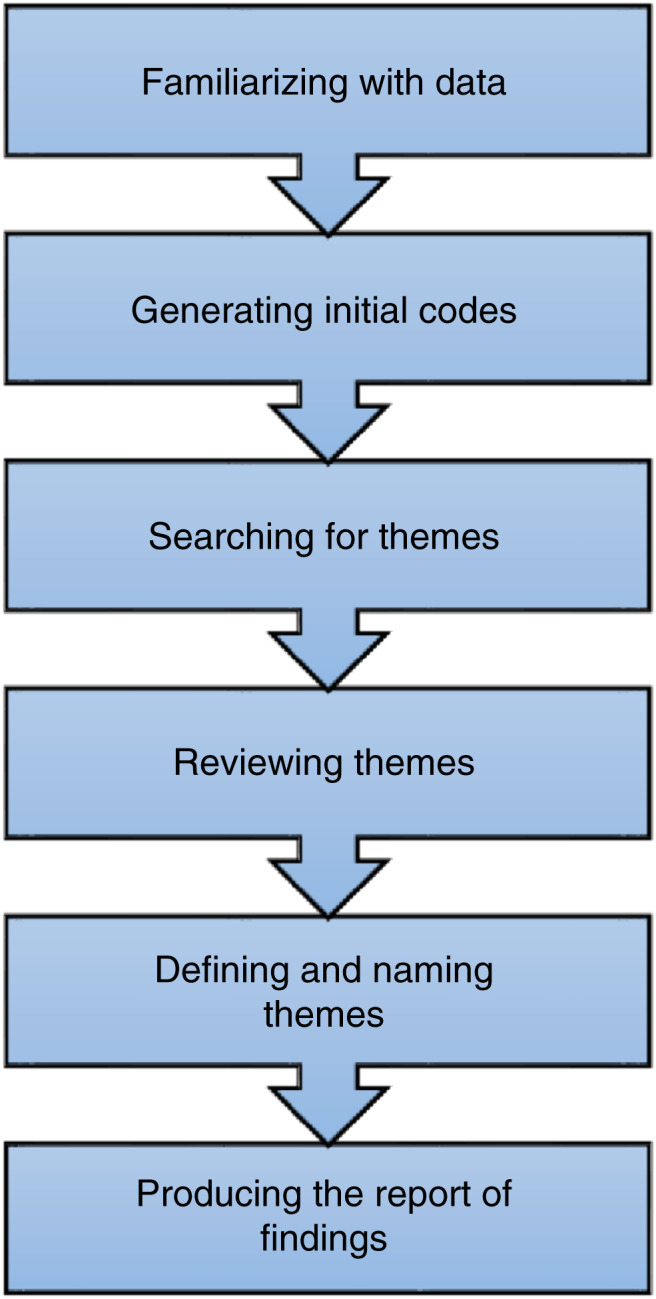
Illustration influenced by Braun and Clarke's six‐step thematic analysis (Braun & Clarke, 2006).

Analysis was conducted using the six‐step approach (illustrated in Figure 1) prescribed by Braun and Clarke (Braun & Clarke, 2006). Thematic analysis is an accessible and flexible theoretical method of qualitative analysis to construct and organize data into themes across a dataset. The analysis was conducted as an abductive analytic process, moving back and forth from data to theorizing, to unfold and create narratives about the phenomenon investigated (Braun & Clarke, 2006).

Analysis was conducted on the transcribed textual data. The data from the first and second interviews for each patient were combined into one analysis to explore the individual patients' personal development and experience over time. First, the authors briefly read and discussed the transcripts of the interviews, focusing on participants' experiences related to the constructs of body ideal, body reality, body presentation and preliminary interpretations. Then the first author conducted the process of coding in NVivo: first, an open coding, followed by an axial coding, looking across data to flesh out key conceptual dimensions and account for variations, leading to the construction of draft themes. NVivo supported this process by making procedures explicit and transparent. Next, the draft was discussed and interpreted by the first and second authors, leading to a novel construction and specification of themes. The analysis was undertaken in Danish, and when final themes were identified, quotes selected to illustrate the themes and subthemes were translated into English. The selected quotes were translated forward and backward by the first author according to quality assurance criteria for accuracy and correct usage of language in medical translation (Karwacka, 2014). Translations were agreed on by the authors. Participant quotes are included in the following presentation of findings to support the analysis.

### 2.6 Ethical considerations

Having received full information about the study aims and the data collection process, all participants provided informed written consent for their participation. Ethical clearance was obtained by the National Committee on Health Research Ethics and the Danish Data Protection Agency (REG‐104‐2021). This study was conducted in accordance with the principles of the Declaration of Helsinki ('World Medical Association Declaration of Helsinki: Ethical Principles for Medical Research Involving Human Subjects', 2013).

## 3 FINDINGS

The analysis resulted in two overall themes, described in detail below: (1) 'Treatment is required for life‐threatening cancer', and (2) 'Striving for a new normal body'. Common to the two themes were patients' feelings of being on a pendulum, reflecting on who they were in the past, their current rationale and transitioning to their life beyond breast cancer with a changed body. Figure 1 shows the conceptual time perspective that was an underlying mechanism throughout the identified themes.

### 3.1 Theme 1: Treatment is required for life‐threatening cancer

This theme encapsulates the finding that participants were confronted with life‐threatening cancer that required immediate treatment, and they were highly dependent on physicians for their treatment, and by extension, for their own future. The theme was further divided into two subthemes: 'The priority is to treat the cancer' and 'Being in the hands of the physicians'.

#### 3.1.1 The priority is to treat the cancer

The subtheme 'The priority is to treat the cancer' was found to be a very strong underlying premise for study participants, as the life‐threatening cancer diagnosis was the overarching fact occupying their minds before thinking of oncoplastic breast surgery. Participants expressed that receiving a cancer diagnosis was like a fatal 'stamp' indicating that they could die without treatment. Living with a cancer diagnosis were described by one participant as: '*One tends to associate cancer with death, like cancer equals death*' (Participant 1). Another participant described their cancer diagnosis saying, '*I was struck by lightning, but I have been lucky to survive so far*' (Participant 7).

The participants experienced difficulty talking about their wishes related to their future breasts and body image. Treating the cancer in a way that would lead to the best possible outcome was their top priority. One participant said:

*The only thing on my mind when I got my diagnosis was for the cancer to disappear and I did not think much about what different kinds of surgery would mean related to femininity or to the choice of surgery in general… I just wanted the breast cancer to go away as soon as possible…* (Participant 3).

For the participants in the diagnosis phase, this prioritization of cancer treatment meant, quite rationally, postponing thoughts about having breasts in the future. However, the participants did express concerns related to the potential long‐term effects of an altered body image, and because of this, they proactively requested oncoplastic breast surgery.

#### 3.1.2 Being in the hands of the physicians

This subtheme captures participants' experiences of dependency during cancer treatment and in relation to the oncoplastic breast surgery, they were offered. The participants who went through chemotherapy stated that the treatment was controlled by their oncologist and that there was a recommended course of treatment to follow to achieve the best possible outcome and recovery. The participants experienced being in the hands of different specialists—breast surgeons, oncologists and plastic surgeons, each of whom had distinct perspectives related to the oncoplastic breast surgery trajectory, which blurred the long‐term plan in participants' minds. This theme was also associated with waiting for chemotherapy to take effect, or waiting to heal from surgery, so that the physician would present the next step in the process of treatment, or 'where to go next.' For example, one participant commented: '*The physicians are in control of my cancer treatment plan… I do not know if I can get the operation while I am in chemotherapy or whether I have to wait until it is finished*' (Participant 1).

Approval for oncoplastic surgery was highly dependent on a patient's condition, such as their body mass index and physical status; not all the participants were candidates for all types of breast reconstruction or correction. One participant offered nuanced insights into how she was in the hands of the physician, stating:

*I do tend to get worried… The last time I got filler in my tissue expander, the physician or surgeon told me that I had three to four months to lose weight if I wanted the final operation… At the same time, that is a completely unfair battle for me because I get antihormones which impede and complicate my weight loss*. (Participant 6).

Participants were dependent on the judgement of physicians, who acted as gatekeepers for treatment and surgical possibilities.

### 3.2 Theme 2: Striving for a new normal body

By their second interviews, most participants had completed their initial chemotherapy or surgical treatment; some had yet to complete certain steps, such as plastic surgery, expanders, corrections or nipple tattoos. The theme 'Striving for a new normal body' captured participants' reflections on future aspects related to their body and self. This theme also illustrated how the participants were dealing with their body at the time of the interviews, and that the process of identifying with a changed body led to different thoughts on the appearance of their breasts and their acceptance of the cosmetic result. The interpretation flowed into three subthemes: 'At least there is something in my bra,' 'Wishing for a plan for my new breasts' and 'Redefining myself after breast cancer'.

#### 3.2.1 At least there is something in my bra

The subtheme 'At least there is something in my bra' relates to patient experiences of their breasts post‐surgery: a reconstructed breast had weight and volume even if it was no longer a natural breast. The participants in the study expressed broad levels of satisfaction with the results of the oncoplastic breast surgery. For example, one participant said:

*I could wish for as many as possible to get the [oncoplastic breast] surgery…. It is a big deal that you still have breasts. They may not look very, you know, but I do think they [the surgeons] have done a good job. At least there is something to fill in the bra, so I guess people will never notice that the breast is not mine* (Participant 1).

However, participants had different perspectives on the importance of physical appearance and aesthetic results from oncoplastic breast surgery. A commonality was that participants were pleased that their reconstructed breast had enough volume to fill a bra, therefore obviating a breast prosthesis. As one participant expressed:

*I had to accept that it [the breast] looks very different than my own breast, but as I have now got the message that the cancer is gone, I feel now that I can expect a decent result that I can live with the rest of my life. I do feel that I am fairly young. Maybe it would be different if I was 80 years old – then it would not be so important… But the breast and shape does imply a femininity which I have always had… Therefore I think it would be much harder for me if they [the breasts] suddenly were not there*… (Participant 7).

Participants also compared the surgical outcome with the alternative of having no breast(s) and reasoned that the volume of the reconstructed breast(s) was better than a flat chest due to mastectomy.

#### 3.2.2 Wishing for a plan for my new breasts

During the second interview, the participants had completed the initial phases of treatment and were increasingly preoccupied with the construction of the new breast(s), identified in the 'Wishing for a plan for my new breasts' subtheme. The participants also expressed a wish for a concrete plan for the reconstruction of their breasts as part of returning to daily life, for example: '*I want to move on with my life now… That is also why I tend to get a bit sceptical [of the reconstruction operation plan]… When can I get the operation, because it stops my work life…?*' (Participant 7). When the participants did not have a clear plan, they expressed insecurity, which could indicate uncertainty about reconstruction:

*I think the plan is on standby for now, they [the physicians] have to see the result. People tell me, like, wow, those [new breasts] are so nice… That might be true, I do not know, and I do not think I can commit to this yet because I do not know if they are finished or if they [the prostheses] are here to stay* (Participant 7).

Another participant also expressed frustration due to a lack of answers to specific questions they had related to their breasts:

*I do not quite know how much volume I can get into the expanders. When I ask, I feel that is it not a subject of importance and that the issue is being easily bypassed because they tell me that we must wait and see how much volume the expander and skin can hold and how the breast shapes, so I listen to that, although I cannot get a concrete answer… To me, it is important if the breasts look how I want or if I just have to fit into their opinion* (Participant 2).

Another participant raised the issue that plans for breast reconstruction should have been discussed at the diagnosis stage, even though participants often focused initially on treatment rather than future breast reconstruction: '*I could have used that when I got the breast cancer diagnosis and I was told that my breast should be removed; someone should have talked to me about the options [related to the breast]—what would have been good for me and what could possibly happen*' (Participant 1).

#### 3.2.3 Redefining a self after breast cancer reconstruction

During the interviews, participants elaborated on what they experienced as a unique situation: dealing with a cancer diagnosis while simultaneously being hopeful about their future. This subtheme was identified as 'Redefining a self after breast cancer reconstruction'. One participant commented on their way of coping with the situation:

*My focus has been to get going after the disease, being me and keeping up the everyday life we used to have. For me it was like ‘get into the game,’ cheer up, take a walk and go to work…* (Participant 5).

Another participant expressed a rather pragmatic way of redefining the body after cancer and reconstructive surgery:

*Looking at my breasts is very extraordinary. Feeling them is extraordinary. I think it is comparable to giving birth, then the body is a completely different universe until it is healed. In the situation, you adjust to the condition, and you mentally retrieve the situation… It is the same with breasts* (Participant 2).

Participants also elaborated on how the trajectory of treatment had not only affected their body but also their personality: *‘I felt that my personality suddenly changed…I lost my hair [due to chemotherapy] and then I had just small breasts and no hair…Now, after the [oncoplastic breast] surgery, the only one that is affected by the truth [of the previous cancer] is me and my naked body’* (Participant 2). This participant's experience in particular illustrates how individual experiences were affected by past diagnoses; how they approached defining a new self; and how they thought about their future, including integrating it with their personal history. They were experiencing a transition.

## 4 DISCUSSION

This study was performed to investigate women's experiences with oncoplastic breast surgery and how the cancer treatment affected their experiences of body image. Two themes were identified: (1) ‘Treatment is required for life‐threatening cancer’, and (2) ‘Striving for a new normal body’. The first theme summarizes how participants focused primarily on treating the cancer; at the same time, they were dependent on physicians for their cancer treatment and surgical plans. The second theme expresses participants' reflections on future aspects related to their body and self, including their postsurgical experiences of breast reconstruction; this theme also reflects the fact that participants actively requested a plan and were striving to redefine themselves.

**FIGURE 2:**
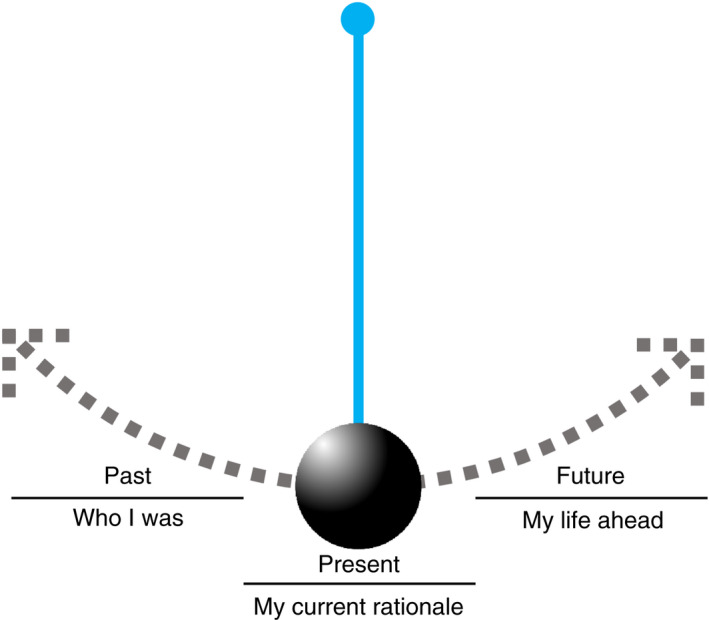
Illustration of participant experiences as a pendulum, as part of transitioning and their altered embodiment.

The themes and subthemes extracted from the interviews with participants illustrate the complex situation women confront when diagnosed with breast cancer, a potentially life‐threatening disease. The interviews indicate that 6 months after diagnosis, participants remained in a condition of acute crisis related to their cancer diagnoses, a finding confirmed by previous research (Berterö & Chamberlain Wilmoth, 2007). Even while managing this state of crisis, participants were striving for a future, one that incorporated their new breasts. This existential situation can be compared with the movement of a pendulum, a metaphor from psychology used with regard to coping strategies in the presence of death (Sand et al., 2009). The pendulum moves between dichotomous dimensions and elicits diverse reactions depending on personal and environmental factors, a metaphor that fits our findings. Participants discussed their past, including how their breasts were before their diagnosis. They expressed the situation as a rationale underlying their life events when expressing their perspectives on the oncoplastic breast surgery. Participants also expressed uncertainty about their future related to the plan for oncoplastic surgery. This pendulum of past, present and future is illustrated in Figure 2. The pendulum effect was also seen in the transitioning, distress and repercussions for participants as they strived to identify with their altered body and self.

In Merleau‐Ponty's phenomenology of perception, changing or replacing parts of the physical body leads to embodiment alterations (Merleau‐Ponty, 2011). The altered body is an integral part of the subjective being, wherein the body influences the mind, and time and transition are required before the altered body is part of the individual's embodiment (Merleau‐Ponty, 2011). Oncoplastic breast surgery affects the whole person: there is a complex relationship between meaning, body and the situation, in which women have their own perceptions of the illness and treatment (Thomas, 2005). Participants in our study experienced that adapting to embodied alterations could be hindered by uncertainty related to future surgical plans and results. Embodiment issues also manifested in how the participants related their self‐image and bodily awareness to embodied normalization; striving to redefine the self was linked to the process of breast reconstruction, having ‘something to put in the bra’.

This perspective of women's embodiment is supported by the embodied body framework by Hopwood and Hopwood, who introduced this framework as an aspect of psycho‐oncology. The authors suggest a multidimensional approach to better recognize and understand the extent, causes and recovery related to altered embodiment; they state that humans cannot untether themselves from the past and that the past will always intrude on the present (Hopwood & Hopwood, 2019b).

Our findings support existing knowledge, affirming that coping with breast reconstruction surgery is complex, with multiple influencing factors. Furthermore, our study demonstrates the embodiment phenomenon among women who are interested in receiving breast cancer reconstruction surgery. The impact of breast reconstruction on participants' embodiment was to support them in redefining their 'new normal'. An interview study by Gershfeld Litvin and Jacoby explored women's experiences when electing to undergo breast reconstruction: women experienced mastectomy as a disability, and their expectations that breast reconstruction would restore their sense of normalcy were not fulfilled. Most women perceived the reconstructed breast mainly as a visual replacement (Gershfeld Litvin & Jacoby, 2017). These findings are consistent with our findings, as participants focused on ‘something to put into the bra’.

In our study, participants requested more information about oncoplastic breast surgery. Access to information was difficult due to varying departmental organizations at different locations in the region and a lack of a standardized care plan or collaboration specifically for oncoplastic surgery. In a 2019 study exploring women's decision‐making on breast reconstruction as a surgical option post‐mastectomy, the authors showed that the choice was negatively influenced by poor acceptance of this surgical procedure among the women. This was possibly due to variation in how physicians communicated treatment options to patients: patients offered breast reconstruction by their primary oncologist were more likely to consider integrating it in their breast cancer treatment plan (Retrouvey et al., 2019). Furthermore, acceptance of breast reconstruction was a complex process that took time, as from the moment of diagnosis to breast reconstruction consideration, survival was prioritized over reconstruction. However, as women realized that breast reconstruction was a way to regain normalcy, this surgical procedure gained acceptance. These findings support our hypothesis that women were initially interested in and focused on the removal of the cancer, potentially because of poor information on surgical procedures from physicians, but also because consideration of oncoplastic surgery might be overwhelming at the time of diagnosis. Another qualitative study by Retrouvey et al. identified that health care professionals do not consistently integrate oncoplastic breast surgery as an option when discussing treatments for breast cancer, affecting delivery between patients (Retrouvey et al., 2020). Likewise, the participants in our study expressed that information on oncoplastic breast surgery was absent at the time of the diagnosis, but at the same time, the participants' focus at that time was mainly on cancer treatment; this is slightly contradictory, with ambiguous implications for what information nurses and physicians should introduce to patients, how and when.

In a survey from 2019, Herring et al. showed considerable variability in women's experiences of their appearance after mastectomy and/or breast reconstruction (Herring et al., 2019). Some felt prepared and emotionally supported while others felt that elements of supportive care were missing. Proposed areas for improvement were preoperative discussions and increased preparation and support for patients, which could potentially contribute to better patient outcomes overall (Herring et al., 2019). A retrospective study from 2019 investigated women's experience of breast reconstruction and their ability to cope (Carr et al., 2019). The authors concluded that as nurses play a pivotal role in providing information to women recovering from breast reconstruction, improving communication channels between nurses and patients is likely to improve patients' experiences of support. Another study showed that nurses can assess women's specific support needs and partner with families to help them understand how best to support women during recovery (Carr et al., 2019), while other studies suggest psychosocial interventions to reduce body image–related distress in women with breast cancer (Brunet et al., 2021; Sebri et al., 2021; Sherman et al., 2018).

Our findings, together with the existing research on women's experiences with oncoplastic reconstruction, indicate doubts concerning the early benefits of reconstruction in general and oncoplastic breast surgery in particular. In some of the literature, oncoplastic breast surgery is presented to women as a procedure that 'cancels out' the mastectomy and facilitates a return to normalcy (Berry et al., 2010; Gilmour et al., 2021; Liu et al., 2018; Macmillan & McCulley, 2016; Riis, 2020; Weber et al., 2020), but this tends to underestimate the range of repercussions reported by women who have undergone the procedure.

Our findings show that women's experiences of oncoplastic breast surgery are complex on the individual level and that the repercussions of the surgery indicate the need for individualized and continuous support from nurses and physicians to support women throughout oncoplastic breast surgery and recovery. Some existing recommendations include increasing interprofessional collaboration at hospitals (Campbell‐Enns et al., 2017; Retrouvey et al., 2020) or deploying advanced nurse specialists in the field of oncoplastic breast surgery specifically to enhance the focus on psychosocial aspects and support women pre‐ and post‐operatively (Spector et al., 2010; Wilson et al., 2013). Furthermore, existing evidence suggests that hope mediates the relationship between self‐compassion about well‐being and distress outcomes in patients (Arambasic et al., 2019; Todorov et al., 2019), indicating that this should be part of specialist breast cancer nurses' psychosocial follow‐up care to help women cope with the impact of breast cancer on their quality of life. This finding is supported by a recent Cochrane systematic review on nurses' support of women with breast cancer which suggests that psychosocial interventions delivered by specialist breast cancer care nurses may improve supportive interventions during diagnosis, treatment and survivorship for women with primary breast cancer (Brown et al., 2021). This evidence clearly suggests that nurse specialists in the field of oncoplastic surgery have an essential role in supporting breast cancer survivors.

The contribution of the present study is that women experience a variety of emotions, sometimes moving from distress to hope for the future, during the process of oncoplastic breast surgery and transitioning to an altered body image. One implication for future practice is that nurses and physicians should carefully inform women about oncoplastic breast surgery, including providing information about the strict guidelines for candidates for oncoplastic breast surgery (Gilmour et al., 2021) and how the treatment is delivered by healthcare professionals from different departments. If women choose oncoplastic breast surgery, nurses and physicians can engage with the women's lived experiences of embodiment and recognize the importance of these experiences concerning the personal recovery process, for instance, through narratives in nursing (Damsgaard et al., 2021).

## 5 STRENGTHS AND LIMITATIONS

To our knowledge, this is the first study to qualitatively investigate the experiences of women undergoing oncoplastic breast surgery influenced by a longitudinal perspective, defined as 'a study design in which data are collected at more than one point in time' (Polit & Beck, 2018). Additional research on embodied perspectives may help to improve nurses' and physicians' understanding of how individuals manage problems or phenomena in illness, leading to better ways of supporting women in oncology settings (Thomas, 2005). Qualitative research of this kind on the oncoplastic breast surgery population can provide additional evidence for developing supportive practices. The current study did not focus on the fact that oncoplastic breast surgery often involves benign contralateral symmetry surgery, which is complex itself and the implications of which should be discussed with patients (Rizki et al., 2013).

The small sample of seven participants providing 14 interviews may be considered a weakness of the study; however, the sampling rationale aimed to engage a small number of individuals experientially familiar with the phenomenon and willing to share their experiences (Braun & Clarke, 2013). The sample size was appropriate to explore the specific research questions on human experiences in oncoplastic breast surgery treatment and the underlying subjective, experiential nature of related phenomena (Braun & Clarke, 2013).

In terms of the quality criteria of validity and generalizability (Braun & Clarke, 2013), the findings in this study are dependent on the specific hospital department from which the participants were recruited, which influences external validity. However, the findings are highly relatable to existing research, which strengthens the trustworthiness of the results (Braun & Clarke, 2013). The findings provide nuanced perspectives from individuals undergoing oncoplastic breast surgery; however, the findings could have been more comprehensive if the study had proceeded as a multi‐centre study. The study findings might also differ if the study was conducted today, as the department has since developed more advanced trajectories for breast cancer treatment, consolidating the departments of plastic and breast surgery.

## 6 CONCLUSION

The contribution of this study is the finding that participants' rationales related to their past, present and future can be compared with a pendulum, transitioning to an altered body image due to the diagnosis of breast cancer and the subsequent oncoplastic breast surgery. At the time of diagnosis, the participating women concentrated on treating cancer, confident in the treatment prescribed by their physicians, and they expressed little interest in the long‐term oncoplastic breast surgery plan. As participants proceeded through treatment and towards surgery, they requested more information about the oncoplastic breast surgery procedures to clarify their future embodied self‐image; they also suggested that this information could have been provided at the time of diagnosis, a suggestion that was at odds with the finding that treatment, rather than reconstruction, was the initial focus.

An implication for future practice is that nurses and physicians caring for women with breast cancer who are candidates for oncoplastic breast surgery need to provide person‐centred care and information throughout the treatment process, from diagnosis and surgery to medical treatment and recovery, to engage with the women's lived experiences of embodiment and body image and to recognize the importance of these experiences in their transitions.

## AUTHOR CONTRIBUTIONS

STH, LWR: Made substantial contributions to conception and design, or acquisition of data, or analysis and interpretation of data; STH, LWR: Involved in drafting the manuscript or revising it critically for important intellectual content; STH, LWR: Given final approval of the version to be published. Each author should have participated sufficiently in the work to take public responsibility for appropriate portions of the content; STH, LWR: Agreed to be accountable for all aspects of the work in ensuring that questions related to the accuracy or integrity of any part of the work are appropriately investigated and resolved.

## FUNDING INFORMATION

This research received no specific grant from any funding agency in the public, commercial or not‐for‐profit sectors.

## CONFLICT OF INTEREST

The authors declare that there is no conflict of interest.

## CLINICAL TRIAL REGISTRATION

Ethical clearance was obtained by the National Committee on Health Research Ethics and the Danish Data Protection Agency (REG‐104‐2021).

### PEER REVIEW

The peer review history for this article is available at https://publons.com/publon/10.1111/jan.15309.

## Data Availability

Data available on request due to privacy/ethical restrictions.

## APPENDIX A

INTERVIEW GUIDES

Interview 1—The time of diagnosis

**Table:**

Themes	Interview questions
Body reality Attention to: Satisfaction with breast size/shape, appearance, pain, physical condition, issues with physical activities, sexuality. Bodily reality immediately after the breast cancer operation. The physical body in the acute disease phase.	1. *How did you experience your health condition and physical body before you got your breast cancer diagnosis? How do you experience your body at this stage after the surgery?* Supportive questions How was your physical and mental status before you got breast cancer? What does it mean for you to live healthily? How is your physical condition? What did you think about your breast size, shape and symmetry before breast surgery? Did you breastfeed? How did that go? Did you have any discomfort in your neck, shoulders or breast area before surgery which caused you pain? Related to your breasts, did you feel comfortable during sexual activity or intimacy? Were you satisfied with your breasts before surgery? Do you feel physically inconvenienced by your breasts now? What are your expectations related to inconvenience and your breasts in the future? What do you think about your breast size, shape and symmetry now? What are your thoughts about intimacy related to the changes in your breasts? Do the physical changes correspond to your expectations? 2*. What are your thoughts about your breast cancer diagnosis?* Supportive questions What was your experience when you got your breast cancer diagnosis? What are your thoughts about the consequences of the diagnosis? Have you thought about the following treatment after the breast surgery? How do you think your breast cancer diagnosis may potentially affect your family/work/social relations? How does the breast cancer diagnosis influence your body image? 3*. Can you describe what you were thinking when you were offered oncoplastic surgery as part of your cancer treatment?* Supportive questions Was your reaction to the offer positive/negative? Why/why not? How did you experience the information you received related to the oncoplastic procedure? Did your relatives, the surgeon or the nurse provide you with information regarding the effects of the oncoplastic surgery? How?
Body ideal Attention to: Female body ideals, the influence of nutrition, societal norms.	4. *Can you tell me about your ideal body? What is important to you?* Supportive questions Thinking of your ideal life, how would you prefer your appearance to be? Is your weight as you would wish? How important is healthy diet to you? What are your thoughts about fashion? How would you describe the *ideal of female beauty in society?* What are your thoughts about societal trends in diet and exercise? How do you think society/media influence our health habits? How important is exercise to you? What do you consider to be bad habits concerning your health?
Patient body presentation Attention to: Satisfaction with reflection in the mirror, physical condition, clothing, comfort in social contexts, equal with other women, attractive (with and without clothing).	5. *Tell me about how you think you present yourself. What perception do you think others have of you?* Supportive questions What are your thoughts about your satisfaction with your appearance with clothes on/in tight clothes/without clothes? What is important to you concerning your clothes/your body appearance? What style do you consider yourself to have/what 'type' are you? Do you present yourself differently at work versus in your spare time? If so: which way do you prefer to present yourself? What did your relatives say about your appearance before and after surgery? What feedback have you received on how you presented yourself before the operation? How important is it to you to be sexually attractive? How do you function in social contexts? What do you think of your body when you're in company with other women? How do you think you present yourself right now, after the operation?

Interview 2—Post surgery

**Table:**

Themes	Interview questions
The patient's body reality Attention to: Satisfaction with size and shape of breasts, appearance, pain, physical condition, problems with physical activity, sexuality The surgeon's ability to inform. Confidence in the situation	1. *How do you consider your health status and your body in physical terms now, six months after the operation?* Supportive questions Do you feel as healthy, i.e. in good health and fit, as you wish to be? Has your perception of being healthy and in good health changed and how? How is your physical shape? What are your thoughts about the size, shape and symmetry of your breasts? What are your thoughts about the overall cosmetic result of the plastic surgery? Have you had or do you still have discomfort in the neck, shoulders or chest area? How does the reconstructed breast feel? What are your thoughts on your satisfaction with your sex life after surgery? How does the cosmetic change in your breast impact your sex life? 2. *What thoughts do you have on your treated breast cancer?* Supportive questions: What physical and mental discomforts have you gone through post breast cancer treatment? How prepared for the discomforts have you felt? What are your thoughts about the information you received about the breast cancer treatment? What do you think about your prognosis? 3. *Describe your reflections on the offer of plastic surgery you were given*. Supportive questions: How was the information given to you about the plastic surgery? Did the explanation from the surgeon reflect the result? Did you have the opportunity to get your questions answered after your discharge from the hospital? How prepared did you feel for the physical impacts of the plastic surgery? How prepared did you feel for pain, tightness of tissue, healing, infection, reduction of physical activity?
The patient's body ideal Attention to: female ideal, nutritional impact, societal norms.	4. *Tell me about your body ideal after your surgery. What do you consider important?* Supportive questions: If your life was ideal, how would you like your appearance to be and has it changed during the cancer trajectory? Is your weight as you wish? How important is what you eat and drink to you and has this changed? What are your thoughts on current fashion trends? Has your perception of the current female ideal changed in connection to your surgery? What style do you consider yourself to have? What does what you eat and drink mean to you and has that changed in connection to your surgery? What does exercising mean to you? Which bad habits concerning your health do you think you have?
The patient's body presentation Attention to: Satisfaction with reflection in the mirror, physical condition, clothing. Feeling comfortable in social contexts, feeling equal to other women and feeling attractive (with and without clothes).	5. *Tell me what you think about your apperance now, six months after your surgery?* Supportive questions: To what degree are you now satisfied your apperance with your clothes on, in tight clothes, and without clothes on? What is important to you concerning your clothes and the apperance of your body, and has this changed? What do those nearest to you say about your appearance? What feedback have you heard about your appearance during the six months after your surgery? How important is it to you to be sexually attractive? How do you function in social contexts? What do you think of your body when you're in company with other women?

## APPENDIX B

CONSOLIDATED CRITERIA FOR REPORTING QUALITATIVE STUDIES (COREQ): 32‐ITEM CHECKLIST

Checklist of items that should be included in reports of interviews and focus groups according to Tong et al., 2007

**Table:**

Section/topic	# Item	Guide questions/description	Reported on page #
Domain 1: Research team and reflexivity	Personal Characteristics		
	Interviewer/facilitator	Which author/s conducted the interview or focus group?	9
	2 Credentials	What were the researcher's credentials? E.g. PhD, MD	8
	3 Occupation	What was their occupation at the time of the study?	8, 9
	4 Gender	Was the researcher male or female?	8
	5 Experience and training	What experience or training did the researcher have?	8
	6 Relationship established	Was a relationship established prior to study commencement?	8
	7 Participant knowledge of the interviewer	What did the participants know about the researcher? e.g. personal goals, reasons for doing the research	12
	8 Interviewer characteristics	What characteristics were reported about the interviewer/facilitator? e.g. Bias, assumptions, reasons and interests in the research topic	8
Domain 2: Study design	Theoretical framework		
	9 Methodological orientation and Theory	What methodological orientation was stated to underpin the study? e.g. grounded theory, discourse analysis, ethnography, phenomenology, content analysis	8, 10, 11
	Participant selection		
	10 Sampling	How were participants selected? e.g. purposive, convenience, consecutive, snowball	9
	11 Method of approach	How were participants approached? e.g. face‐to‐face, telephone, mail, email	9
	12 Sample size	How many participants were in the study?	9
	13 Non‐participation	How many people refused to participate or dropped out? Reasons?	9
	Setting		
	14 Setting of data collection	Where was the data collected? e.g. home, clinic, workplace	9–10
	15 Presence of non‐participants	Was anyone else present besides the participants and researchers?	10
	16 Description of sample	What are the important characteristics of the sample? e.g. demographic data, date	Table 1
	Data collection		
	17 Interview guide	Were questions, prompts, guides provided by the authors? Was it pilot tested?	Appendix A
	18 Repeat interviews	Were repeat interviews carried out? If yes, how many?	10
	19 Audio/visual recording	Did the research use audio or visual recording to collect the data?	10
	20 Field notes	Were field notes made during and/or after the interview or focus group?	‐
	21 Duration	What was the duration of the interviews or focus group?	10
	22 Data saturation	Was data saturation discussed?	10
	23 Transcripts returned	Were transcripts returned to participants for comment and/or correction?	‐
Domain 3: Analysis and findings	Data analysis		
	24 Number of data coders	How many data coders coded the data?	11–12
	25 Description of the coding tree	Did authors provide a description of the coding tree?	‐
	26 Derivation of themes	Were themes identified in advance or derived from the data?	11–12
	27 Software	What software, if applicable, was used to manage the data?	11–12
	28 Participant checking	Did participants provide feedback on the findings?	‐
	Reporting		
	29 Quotations presented	Were participant quotations presented to illustrate the themes/findings? Was each quotation identified? e.g. participant number	13‐19
	30 Data and findings consistent	Was there consistency between the data presented and the findings?	‐
	31 Clarity of major themes	Were major themes clearly presented in the findings?	‐
	32 Clarity of minor themes	Is there a description of diverse cases or discussion of minor themes?	‐
